# Integrative analyses of metabolome and transcriptome reveal differences in terpenoids and identify the genes involved in the synthesis of terpenoids in *Magnoliae Flos* from different varieties

**DOI:** 10.3389/fpls.2026.1894544

**Published:** 2026-08-26

**Authors:** Rui Ma, Hongdan Liu, Weimeng Feng, Xiuyu Liu, Mengge Qin, Xinjing Gui, Suiqing Chen

**Affiliations:** 1 School of Pharmacy, Henan University of Chinese Medicine, Zhengzhou, China; 2 Henan Key Laboratory of Chinese Medicine Resources and Chemistry, Zhengzhou, Henan, China; 3 Henan University of Chinese Medicine, Collaborative Innovation Center of Research and Development on the Whole Industry Chain of Yu-Yao, Zhengzhou, Henan, China

**Keywords:** *Magnoliae Flos*, metabolic regulatory network, metabolome, terpenoids, transcriptome

## Abstract

**Introduction:**

*Magnoliae Flos (MF)*, the dried flower buds of *Magnolia denudata* Desr., *Magnolia biondii* Pamp., and *Magnolia sprengeri* Pamp., is a crucial East Asian medicinal herb used to treat allergic rhinitis and other ailments. However, the molecular basis for its quality differences remains unclear.

**Methods:**

This study integrates GC-MS-based volatile metabolomics, high-throughput RNA sequencing, and RT-qPCR validation to elucidate the terpenoid metabolic differences and their regulatory mechanisms among the three *MF* varieties.

**Results:**

The metabolomic analysis screened 55 differential terpenoids (15 key markers) that distinguish the *MF* varieties, with terpenoids being the primary metabolic category and the *M. sprengeri* vs. *M. biondii* group exhibiting the most differential metabolites. The transcriptomic analysis revealed *TPS26* as key candidate genes involved in monoterpene synthesis, while IMPMBI2G0000034954 and IMPMBI2G0000034957 were identified as candidate genes for sesquiterpene synthesis. The expression abundances of these genes exhibited significant linear correlations with the accumulation levels of differential terpenoids, though such coordinated variation does not confirm direct causal regulation.

**Discussion:**

Collectively, this work reveals the molecular basis of terpenoid diversity in *MF*, and provides theoretical references for germplasm discrimination, quality evaluation and genetic improvement of medicinal magnolia resources.

## 1 Introduction

*Magnoliae Flos (MF)*, commonly known as “Xin-yi” in China, refers to the dried flower buds of specific *Magnolia* species harvested in late winter or early spring prior to blooming (Gil et al., 2022; Yi et al., 2025). For centuries, this medicinal herb has been widely used in traditional medicine systems across East Asia for treating allergic rhinitis, headaches, sinusitis, rheumatoid arthritis, and asthma (Shen et al., 2008; Phan et al., 2022). Among the various *Magnolia* species, *Magnolia denudata* Desr., *Magnolia biondii* Pamp., and *Magnolia sprengeri* Pamp. are officially recognized as authentic medicinal sources in the Pharmacopoeia of the People’s Republic of China (Bhuia et al., 2023; Liu et al., 2024; China, 2025). The increasing demand for *MF* in both traditional and modern medicine has highlighted the need to clarify the chemical basis of its efficacy and the molecular mechanisms underlying the quality differences among various medicinal varieties (Huang et al., 2024).

The primary constituents of *MF* are generally categorized into lipid-soluble and water-soluble components, with lipid-soluble compounds representing the predominant bioactive fractions (Zhou et al., 2016; Yi et al., 2025). Lipid-soluble constituents are rich in volatile oils and lignans, which play a crucial role in determining the medicinal efficacy and quality of *MF.* Volatile oils exhibit significant bioactivities (Phan et al., 2022), with over 90% of their essential components being terpenoids (Shen et al., 2008). Pharmacological studies have extensively elucidated the mechanisms of these terpenoids, highlighting that their anti-inflammatory effects are predominantly mediated by monoterpenes such as 1,8-cineole (Gil et al., 2023; Guo et al., 2024). Furthermore, key monoterpenes—including α-pinene, β-pinene, camphor, and linalool—have demonstrated therapeutic potential against respiratory diseases like allergic rhinitis and asthma (Utegenova et al., 2018; Gibbs, 2019), alongside potent antimicrobial properties (Aćimović et al., 2020; Liu et al., 2020).

Terpenoid biosynthesis in plants relies on two evolutionarily conserved pathways: the mevalonate (MVA) pathway in the cytoplasm and the 2-C-methyl-D-erythritol 4-phosphate (MEP) pathway in plastids (Mitsuhashi and Abe, 2018), which produce C5 precursors that subsequently formed prenyl diphosphates (Fierascu et al., 2021; Wang et al., 2025b). These intermediates are subsequently condensed into prenyl diphosphate substrates—namely geranyl diphosphate (GPP), farnesyl diphosphate (FPP), and geranylgeranyl diphosphate (GGPP)—which serve as direct substrates for terpene synthases (*TPS*) to form the core skeletons of mono-, sesqui-, and diterpenoids, respectively (Kong et al., 2022; Wang et al., 2022; Gong et al., 2024). Hydroxymethylglutaryl-CoA reductase (*HMGR*) and 1-deoxy-D-xylulose 5-phosphate synthase (*DXS*) serving as rate-limiting enzymes initiating these pathways (Wang et al., 2022; Khodadadi et al., 2023). Beyond these initial steps, the remarkable structural diversity and species-specific accumulation of terpenoids are largely determined by the substrate specificity of *TPS* enzymes and further shaped by tailoring enzymes, including cytochrome P450 monooxygenases (CYPs), glycosyltransferases (GTs), and acyltransferases (ACTs), which introduce hydroxyl, glycosyl, or acyl moieties to generate bioactive derivatives (Feng et al., 2011; Xiaoyan et al., 2019; Yanbin et al., 2022).While *TPS* gene families have been shown to drive flavor diversity in tea plants (Jiang et al., 2023; Wei et al., 2025), However, the species-specific variations in terpenoid profiles and the molecular mechanisms governing their biosynthesis in *MF* remain inadequately understood.

In recent years, integrated transcriptomic and metabolomic analyses have emerged as powerful approaches for elucidating the molecular basis of secondary metabolite biosynthesis in medicinal plants (Li, 2024; Liu et al., 2025). This combined approach allows for the simultaneous identification of differential metabolites and their corresponding regulatory genes, thereby providing comprehensive insights into metabolic pathways and their transcriptional regulation (Tan et al., 2024; Pan et al., 2025; Wang et al., 2025a). For example, similar multi-omics strategies have successfully elucidated the mechanisms of *TPSs* in *Jerusalem artichoke* (Wang et al., 2022), *Rhododendron fortunei Lindl* (Yi et al., 2024), *Lonicera macranthoides* (Zeng et al., 2025), Camellia sinensis (Wei et al., 2025), lychee (Liu et al., 2025) and *Bidens alba* (Wang et al., 2025b), thereby facilitating the discovery of key genes and metabolic networks. Furthermore, current research on the terpenoid components of *MF* has covered aspects such as component identification, and some pharmacological mechanisms. However, the molecular regulatory mechanisms governing the synthesis and accumulation of terpenoid compounds remain unclear. The core driver genes responsible for the differential formation of terpenoids from different species have yet to be identified, which provides a crucial direction for future studies on the biosynthetic pathways of terpenoids.

**Figure 1:**
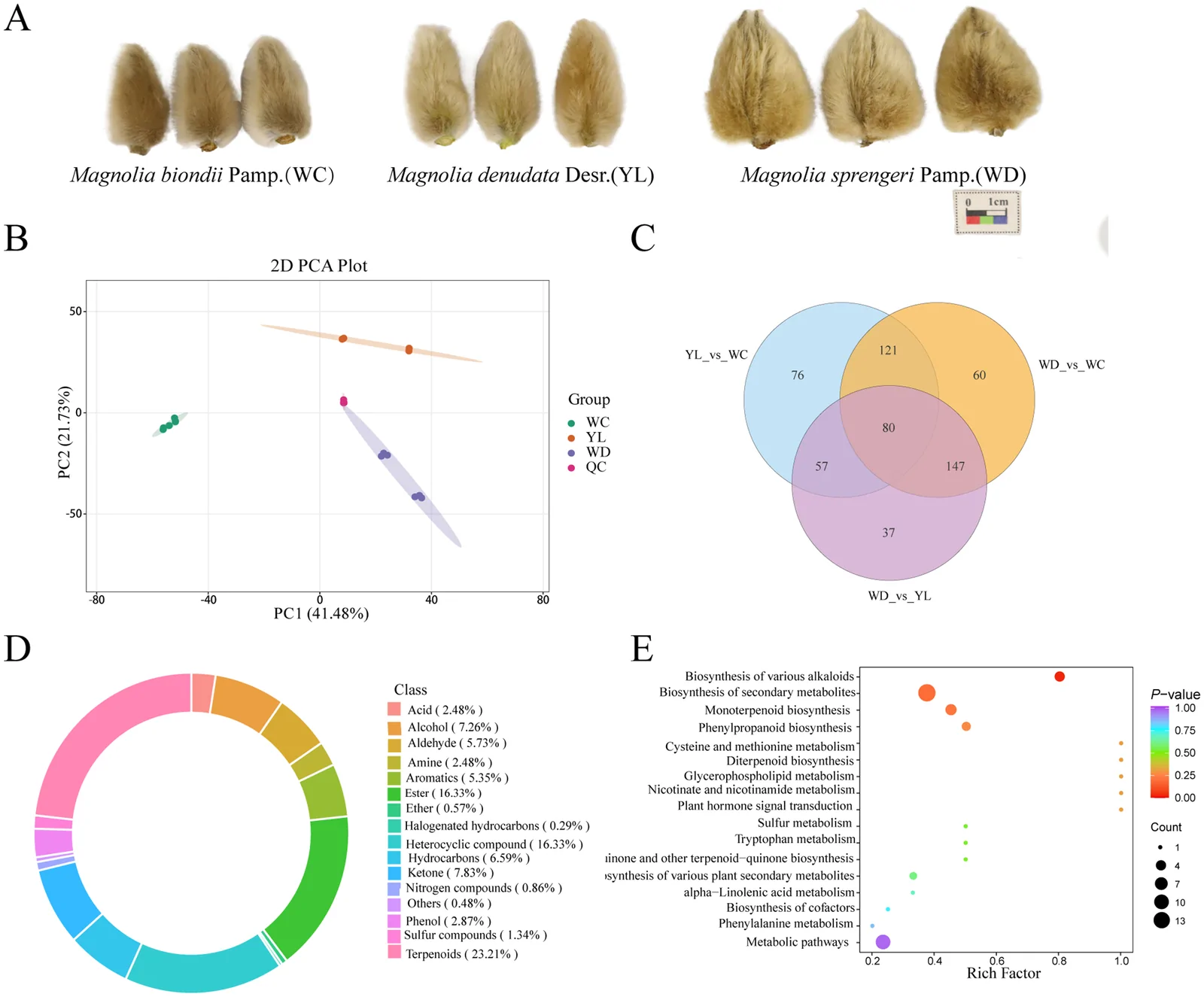
Widely targeted metabolomic profiling of 3 *MF* varieties. WC: *Magnolia biondii* Pamp., YL: *Magnolia denudata* Desr., WD: *Magnolia sprengeri* Pamp. The names and abbreviations corresponding to **Figures 1**-**6** are the same as above. **(A)** The morphological characteristics of flower buds from 3 *MF* varieties. Scale bars = 1 cm. **(B)** Principal component analysis (PCA) of metabolite profiles. **(C)** Venn analysis of metabolites in WC, YL, and WD comparison groups. **(D)** Classification and proportion of identified metabolites. **(E)** KEGG pathway enrichment.

The study focused on three varieties of *MF*: *M. biondii*, *M. denudate*, and *M. sprengeri* (**Figure 1A**). We employed gas chromatography-mass spectrometry (GC-MS)-based volatile metabolomics to characterize terpenoid profiles, complemented by high-throughput RNA sequencing to identify differentially expressed genes (DEGs) associated with terpenoid biosynthesis. Integrated analyses of metabolomics and transcriptomics data, validated through real-time quantitative PCR (RT-qPCR), were conducted to elucidate terpenoids differences among the three *MF* varieties. The aim is to identify key genes and regulatory networks involved in terpene synthesis, thereby providing molecular evidence for the identification, quality control, and genetic improvement of authentic *MF* varieties. The findings will serve as practical references for the sustainable development and utilization of *MF* resources.

## 2 Materials and methods

### 2.1 Plant materials and treatment

The experimental materials of this study consist of fresh flower buds from three different varieties of *MF*. Samples were collected from Nanzhao County in Nanyang City, Henan Province; Xi’an City, Shanxi Province; and Hanzhong City, Shanxi Province (**Supplementary Table 1**). Each group of samples included six biological replicates, with fresh flower buds of similar size and growth randomly selected and labeled as WC1−WC6 (*Magnolia biondii* Pamp.), YL1−YL6 (*Magnolia denudata* Desr.), and WD1−WD6 (*Magnolia sprengeri* Pamp).

### 2.2 GC-MS-based metabolomic analysis

Materials were harvested, weighted, immediately frozen in liquid nitrogen, and stored at -80 °C until needed. Samples were ground to a powder in liquid nitrogen. 500 mg (1 mL) of the powder was transferred immediately to a 20 mL head-space vial (Agilent, Palo Alto, CA, USA), containing NaCl saturated solution, to inhibit any enzyme reaction. The vials were sealed using crimp-top caps with TFE-silicone headspace septa (Agilent). At the time of SPME analysis, each vial was placed in 60 °C for 5 min, then a 120 µm DVB/CWR/PDMS fiber (Agilent) was exposed to the headspace of the sample for 15 min at 60 °C.

After sampling, desorption of the VOCs from the fiber coating was carried out in the injection port of the GC apparatus (Model 8890; Agilent) at 250 °C for 5 min in the splitless mode. The identification and quantification of VOCs were carried out using an Agilent Model 8890 GC and a 7000E mass spectrometer (Agilent), equipped with a 30 m × 0.25 mm × 0.25 μm DB-5MS (5% phenyl-polymethylsiloxane) capillary column. Helium was used as the carrier gas at a linear velocity of 1.2 mL/min. The injector temperature was kept at 250 °C. The oven temperature was programmed from 40 °C (3.5 min), increasing at 10 °C/min to 100 °C, at 7 °C/min to 180 °C, at 25 °C/min to 280 °C, hold for 5 min. Mass spectra was recorded in electron impact (EI) ionisation mode at 70 eV. The quadrupole mass detector, ion source and transfer line temperatures were set, respectively, at 150, 230 and 280 °C. Selected ion monitoring (SIM) mode was used for the identification and quantification of the analytes.

Prior to statistical analysis, missing abundance values of low-concentration undetectable VOCs were imputed following the default MetaboAnalyst workflow (Pang et al., 2022), where each missing value was replaced with one-fifth of the minimum detectable signal of the corresponding metabolite; all annotated VOCs corresponded to independent chromatographic peaks with distinct diagnostic ion pairs, even if they carried identical imputed values. Raw GC-MS data were processed with Agilent MassHunter for peak alignment, spectral deconvolution and quantification, and VOC identification was conducted by matching mass fragmentation profiles, specific qualitative/quantitative ion pairs and proprietary retention indices against the internal metabolite database of Wuhan Metware Biotechnology Co., Ltd.

### 2.3 Transcriptome analysis

Total RNA was extracted from fresh flower buds using the ethanol precipitation method and the CTAB-PBIOZOL method. The successfully extracted RNA was dissolved in 50 µL of DEPC-treated water. Subsequently, the total RNA was identified and quantified using a Qubit fluorometer and a Qsep400 high-throughput biofragment analyzer. After sequencing, raw reads containing adapters, low quality, and those with more than 10% N or with bases of quality value Q10 accounting for over 50% of the entire read were removed to obtain high-quality clean reads. Clean reads were mapped to the published *Magnolia biondii* reference genome assembly Mbi2.fa (available at https://www.bic.ac.cn/IMP/#/Download) via HISAT2 (Kim et al., 2015). StringTie (Pertea et al., 2015) is used to assemble the reads into transcripts. Transcript expression abundances were quantified as fragments per kilobase of transcript per million mapped fragments (FPKM) for differential expression analysis and most visualization plots. Transcripts per million reads (TPM) were additionally calculated and adopted exclusively for cross-sample correlation analysis with RT-qPCR data. Differential expression analysis was performed using DESeq2 software, and multiple hypothesis testing correction of *P* values was conducted using the Benjamini-Hochberg (BH) method to obtain the false discovery rate (FDR). Additionally, enrichment analyses of Gene Ontology (GO) and Kyoto Encyclopedia of Genes and Genomes (KEGG) were implemented for filtered genes via the ClusterProfiler R package (Wu et al., 2021). Fisher’s exact test was adopted as the statistical approach, with the significance threshold set at FDR < 0.05 following Benjamini–Hochberg (BH) multiple testing correction.

### 2.4 Data processing and statistical analysis

Principal component analysis (PCA) and orthogonal pro­jections to latent structures discriminant analysis (OPLS-DA) were performed on metabolomic and transcriptomic datasets through the PRCOMP function in R (https://www.r-project.org). For the analysis of metabolite levels and gene expression, hierarchical clustering and heatmap visualization were carried out using the PHEATMAP package in R. The ideal number of clusters for the metabolites was determined with the help of the factoextra package in R. Co-expression clustering for genes and metabolites was conducted using the Metware Cloud, an accessible online data analysis platform (https://cloud.metware.cn). The Pearson correlation coefficients (PCC) between genes and metabolites were computed utilizing the Cor package in R.

### 2.5 Cojoint analysis of the transcriptome and metabolome

Based on gene expression and metabolite content data, differential metabolites and differential genes were functionally annotated against the KEGG database. To identify shared biosynthetic pathways, pathway over-representation analysis was performed​ using the entire set of genes annotated to KEGG in the present study as the background gene set. This analysis utilized the hypergeometric test to determine significantly enriched pathways, with *P*-values corrected for multiple testing using the BH method. Pearson correlation coefficients (*r*) were calculated between the log_2_ - transformed fold changes of DEGs and differential metabolites (threshold for significance: *P* < 0.05, calculated via two - tailed Student’s *t*-test).

### 2.6 Transcription factor analysis

Based on the transcriptome sequencing results, genes were classified and statistically analyzed according to their transcription factor families. Subsequently, the encoded sequences were subjected to predictive analysis using the plant transcription factor database PlantTFDB (http://planttfdb.gao-lab.org) to determine the regulatory relationships between transcription factors and differentially expressed genes.

### 2.7 RT-qPCR validation

Eighteen key genes involved in terpenoid biosynthetic pathways were selected for quantitative real-time PCR (RT-qPCR) validation. Total RNA was reverse transcribed to synthesize first-strand cDNA using the YALEPIC^®^ All-in-RT MasterMix for qPCR with gDNA off (YPM51341), following the man­ufacturer’s instructions. Gene-specific primers were designed using Primer Premier 5 (**Supplementary Table 2**) (Wang et al., 2017). TEF was used as the internal reference (Kou et al., 2017). Quantitative PCR was performed with the YALEPIC^®^ Universal SYBR Green qPCR MasterMix (YQ51003). All experiments were conducted in triplicate, and relative mRNA expression levels were calculated using the threshold cycle (2^−ΔΔCt^) method (Zhao et al., 2021). The data results are presented as mean ± standard deviation. Variance analysis was performed using SPSS v20.0 software (Yang et al., 2022), and graphs were generated with Origin2019 (v9.6.0.172).

## 3 Results

### 3.1 Metabolomic profiling of 3 *MF* varieties

To investigate the metabolic differences among different species of *MF*, a widely targeted metabolomics approach was employed to systematically analyze three varieties: *M. biondii* (WC), *M. denudata* (YL), and *M. sprengeri* (Bhuia et al.) (**Figure 1A**). A total of 1048 volatile organic compounds (VOCs) were annotated for subsequent statistical analyses. This study adopted a broad-scale volatile screening workflow rather than absolute confirmatory identification of all detected compounds. Principal Component Analysis (PCA) analysis was performed on the total metabolite samples of *MF*. PC1 (41.48%) and PC2 (21.37%) collectively explained the significant separation of the 3 *MF* varieties at the metabolic level. The clustering results of QC samples indicated that the experimental results were stable and reliable (**Figure 1B**).

Venn analysis of metabolites in the WC, YL, and WD comparison groups revealed distinct numbers of differential metabolites: 408 in WD vs. WC, 334 in YL vs. WC, and 321 in YL vs. WD, indicating that WD vs. WC exhibited the highest number of differential metabolites, while YL vs. WD displayed the least (**Figure 1C**). Metabolite classification statistics indicated that a total of 1,048 VOCs were detected. Among these, Terpenoids, Esters, Heterocyclic compounds, Ketones, and Aromatics constituted a significant proportion, accounting for 23.21%、16.33%、16.33%、7.83%、, and 5.35% respectively, with terpenoids being the most predominant (**Figure 1D**). Based on the KEGG database information, functional and classification annotation of the identified metabolites was performed to understand the functional characteristics and classification of different metabolites. The results showed that the differential metabolites of the 3 *MF* varieties were mainly enriched in pathways such as Biosynthesis of secondary metabolites, Metabolic pathways, Monoterpenoid biosynthesis, and Biosynthesis of various alkaloids (**Figure 1E**).These findings comprehensively reveal the metabolic characteristics of the 3 *MF* varieties, providing a theoretical basis for further research on the accumulation patterns of terpenoids.

### 3.2 Metabolic differences of terpenoids of 3 *MF* varieties

**Figure 2:**
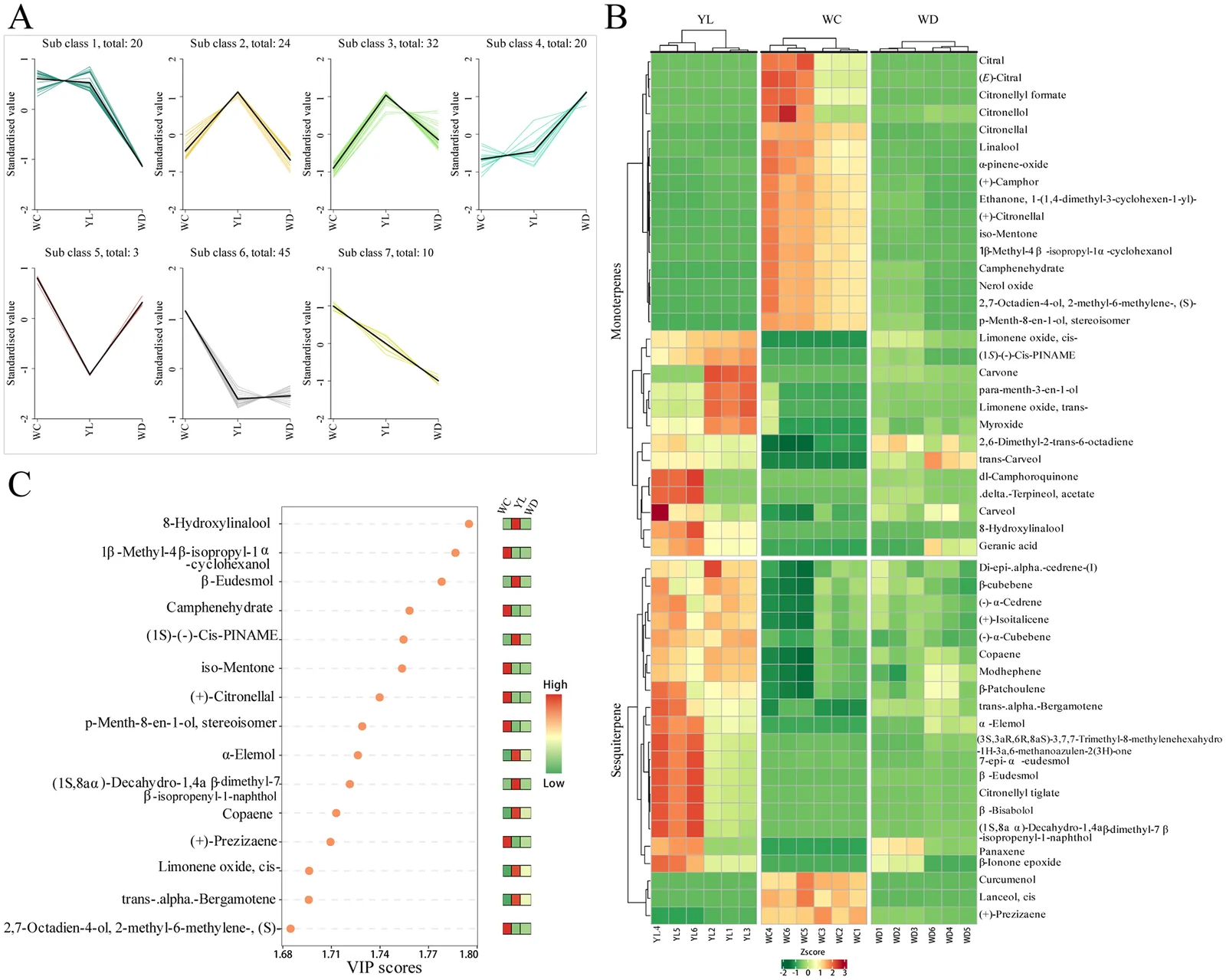
Differential terpenoid metabolites of 3 *MF* varieties. **(A)** K-means clustering of terpenoid metabolites. **(B)** Heat map of terpenoids accumulation patterns. **(C)** Top 15 differential terpenoid metabolites in different comparison groups.

Terpenoids are the major class of bioactive constituents in *MF* (Shen et al., 2008). To better reveal the abundance distribution characteristics of terpenoid metabolites in the 3 *MF* varieties, K-means cluster analysis was performed on the relative contents of identified terpenoid compounds (**Figure 2A**). The results showed that 184 differential terpenoid metabolites were classified into 7 subclasses (**Supplementary Table 3**), with 20, 24, 32, 20, 3, 45, and 10 metabolites assigned to each subclass, respectively. Each exhibiting distinct variety-specific accumulation patterns: Subclass 2 and 3 were mainly enriched in YL, with their metabolites primarily being sesquiterpenoids; Subclass 1, 5, 6 and 7 were mainly enriched in WC, with their metabolites primarily being monoterpenoids; Subclass 4 and 5 were mainly enriched in WD, with their metabolites primarily being sesquiterpenoids. This clustering result visually reflects the variety-specific distribution patterns of terpenoid metabolites.

OPLS-DA was conducted for all comparisons, and a permutation test was performed to verify the reliability of the developed model. Based on the OPLS-DA analysis (**Supplementary Figure 1**, **S2**), with screening criteria set as VIP≥1.5, |log_2_FoldChange| ≥ 2, and FDR≤ 0.01. A total of 50 significantly differential metabolites (DAMs) were identified, including 29 monoterpenes and 21 sesquiterpenes (**Supplementary Table 4**). Most of the sesquiterpenoid DAMs, such as α-elemol, β-cubebene, and β-eudesmol, exhibited orange high-abundance clustering in YL. In contrast, monoterpenoid DAMs like linalool, (+)-camphor, citronellol, and citral showed higher accumulation in WC, while carveol, myroxide, and (+)-limonene oxide displayed higher accumulation in YL. The expression levels of most DAMs in WD were relatively low (**Figure 2B**). Further screening identified 15 terpenoid DAMs with more significant differences in the 3 *MF* varieties, with screening criteria set as VIP≥1.7, |log_2_FoldChange| ≥ 2, and FDR≤ 0.01. Among them, the top five compounds 8-hydroxylinalool, (1β-Methyl-4β-isopropyl-1α-cyclohexanol, β-eudesmol, camphenehydrate, (1S)-(-)-Cis-PINAME)and iso-Mentone) exhibited VIP scores >1.75 (**Figure 2C**), providing important references for subsequent screening of key differential terpenoid components and analysis of their biosynthesis mechanisms.

### 3.3 Transcriptome-based analysis of differential gene expression in 3 *MF* varieties

To explore the potential reasons for the differences in metabolite accumulation of the 3 *MF* varieties, we aimed to investigate the gene expression changes related to terpenoid metabolism at the transcriptional level. Sequencing was performed on samples from three varieties of *MF*. A total of 166.49 GB of clean data was generated, with an average Q30 base ratio of 96.17%. (**Supplementary Table 5**).

**Figure 3:**
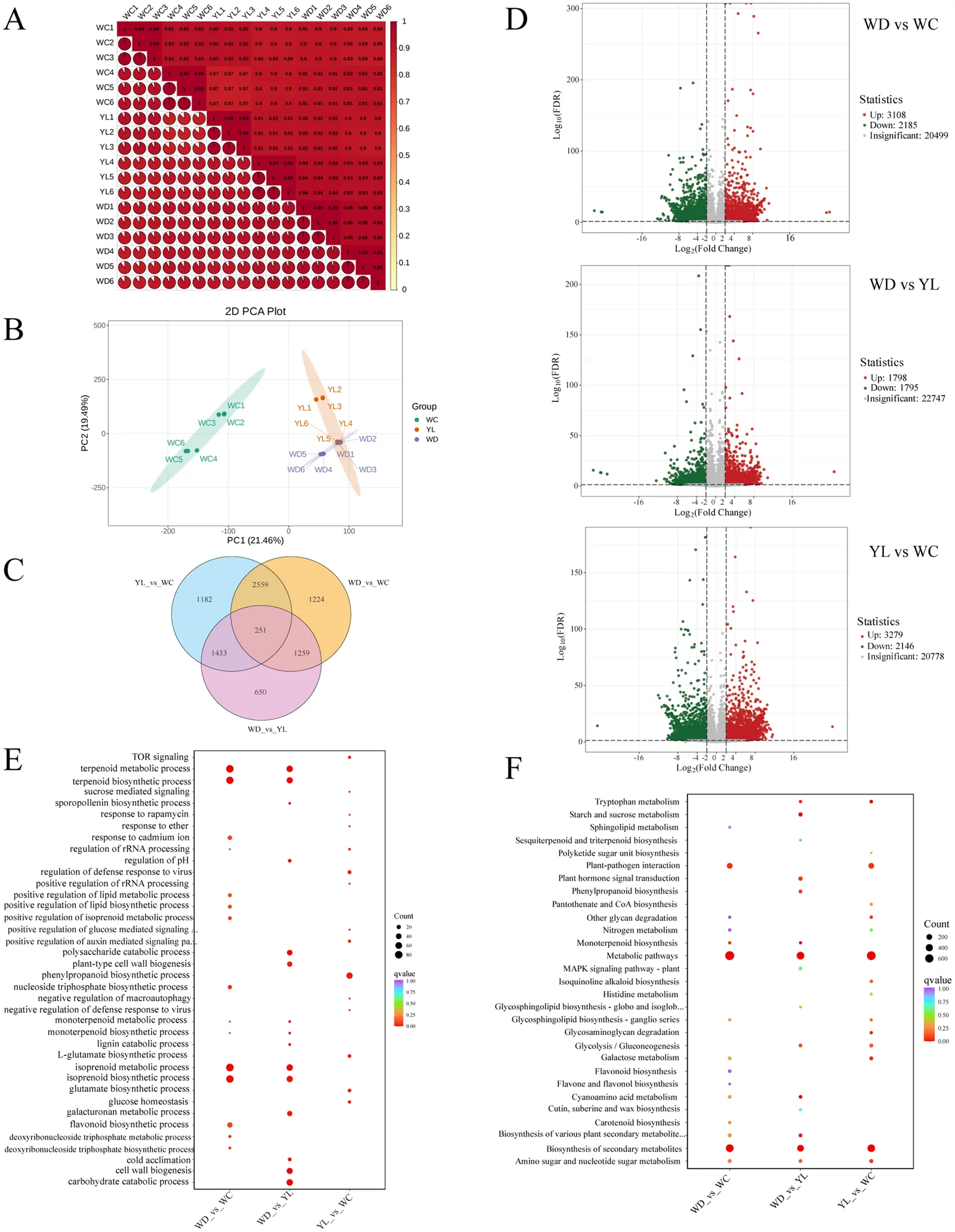
Expression patterns of differentially expressed genes. **(A)** Pearson correlation heatmap of gene expression levels across all transcriptome samples. **(B)** Two-dimensional PCA plot illustrating global transcriptional divergence among WC, YL and WD cultivars. **(C)** Venn diagram. **(D)** Volcano plot of the DEGs in different comparison groups. **(E)** GO pathway enrichment. **(F)** KEGG pathway enrichment.

Pearson correlation analysis of gene expression profiles revealed that the correlation coefficients within each group were all higher than 0.9, indicating reliable biological replicates and stable transcriptome sequencing data. The correlation coefficients between different cultivars ranged from 0.80 to 0.90, reflecting their close genetic backgrounds (**Figure 3A**). PCA results indicated that PC1 and PC2 together explained 40.95% of the total variation (**Figure 3B**). Samples of WC, YL and WD formed three distinct clusters separately, with tight aggregation of biological replicates inside each group. Obvious transcriptional differentiation was observed across cultivars, which validated the rationality of grouping for subsequent differentially expressed gene screening.

Based on this, differential gene expression analysis was performed using DESeq2, with the screening criteria set at |log_2_FoldChange|≥2 and FDR ≤ 0.05. Venn diagram analysis (**Figure 3C**) revealed 251 common differentially expressed genes (DEGs) across all three control groups. A total of 5,293 DEGs were detected in the WD vs. WC comparison group, including 3,108 upregulated genes and 2,185 downregulated genes. In the WD vs. YL comparison group, 3,593 DEGs were identified, comprising 1,798 upregulated genes and 1,795 downregulated genes The YL vs. WC comparison group showed 5,425 DEGs, with 3,279 upregulated genes and 2,146 downregulated genes (**Figure 3D**). To further explore the biological functions of these DEGs, GO and KEGG pathway enrichment analyses were performed on all comparison groups. GO pathway enrichment analysis revealed that the DEGs were primarily enriched in isoprenoid metabolic process (81 and 55 DEGs, *q* value < 0.05, in WD vs. WC and WD vs. YL, respectively; not significantly enriched in YL vs. WC), terpenoid metabolic process (76 and 53 DEGs, *q* value < 0.05, in WD vs. WC and WD vs. YL, respectively; not significantly enriched in YL vs. WC), and terpenoid biosynthetic process (72 and 49 DEGs, *q* value < 0.05, in WD vs. WC and WD vs. YL, respectively; not significantly enriched in YL vs. WC) (**Figure 3E**). KEGG pathway enrichment analysis showed that the DEGs were mainly concentrated in biosynthesis of secondary metabolites (500, 484, and 326 DEGs, *q* value < 0.05, in WD vs. WC, YL vs. WC, and WD vs. YL, respectively) (**Figure 3F**). These results highlight the complexity of the regulatory mechanisms governing terpenoid metabolism, indicating that the specific regulatory logic requires further systematic resolution.

### 3.4 Correlation analysis between DAMs and DEGs

**Figure 4:**
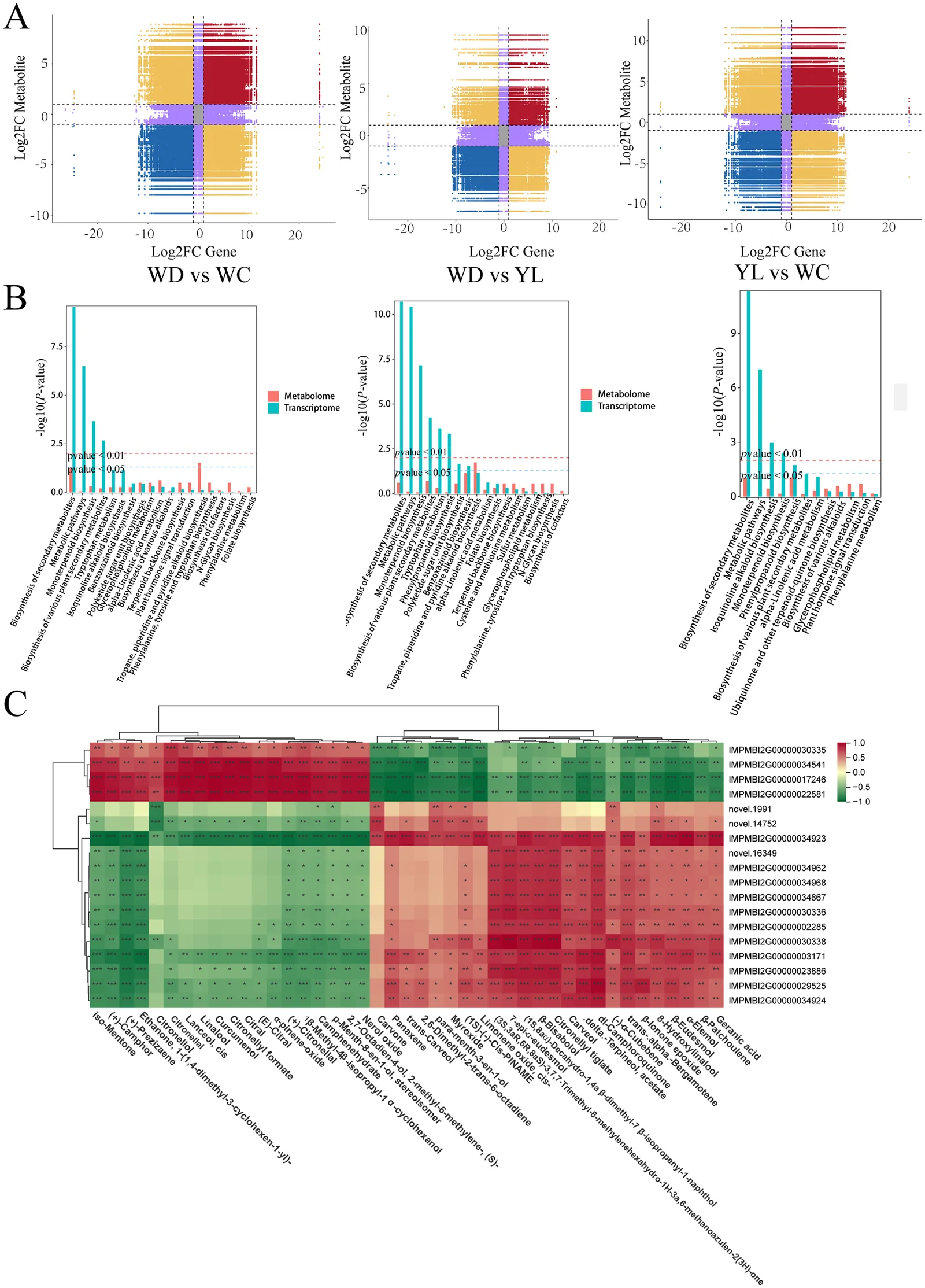
Correlation analysis between DAMs and DEGs. **(A)** Nine-quadrant plot. **(B)** Combined KEGG enrichment analysis. **(C)** Correlation analysis of terpenoids and terpene synthesis genes in 3 *MF* varieties.

Differentially expressed genes (DEGs) directly or indirectly regulate the synthesis and accumulation of metabolites by encoding key enzymes in metabolic pathways, thereby leading to metabolic differences among different plant varieties. This transcriptome-metabolome nine-quadrant plot (**Figure 4A**) displays the trends of gene expression (log_2_FC on the horizontal axis) and metabolite accumulation (log_2_FC on the vertical axis) in comparisons among three varieties. The dense clustering of data points in the red (up-regulated) and blue (down-regulated) quadrants reveals a correlation between transcriptional changes and metabolite accumulation. In WD vs. YL, the wider red area corresponds to the smallest differences in genes and metabolites between WD and YL. In the YL vs. WC comparison, the denser distribution of blue and reddots indicates the most significant differences in genes and metabolites between YL and WC.

The KEGG pathway enrichment bar chart further confirms this observation (**Figure 4B**). Across all comparisons, the differentially expressed genes and metabolites were significantly enriched in metabolism-related pathways, particularly the biosynthesis of secondary metabolites and monoterpenoid biosynthesis. In contrast, pathways related to basic primary metabolism were less abundant, highlighting that metabolic specialization among the 3 *MF* varieties focuses more on secondary metabolism rather than large-scale differentiation in primary nutritional metabolism. This implies that the regulatory relationship between transcriptional control and metabolite accumulation is intricate, necessitating deeper investigation into the precise logic governing varietal differences. To further validate this, we conducted correlation analysis on DAMs and DEGs in the terpenoid pathway (|r| > 0.8 and *P* < 0.05) (**Figure 4C**; **Supplementary Table 6**). Monoterpene compounds such as linalool, (+)-camphor, α-pinene-oxide, and (*E*)-citral showed significant positive correlations with key terpene synthesis genes including *CHLP* (IMPMBI2G00000034541), *G10H* (IMPMBI2G00000022581), and *DXS* (IMPMBI2G00000028900). Meanwhile, sesquiterpene compounds like β-eudesmol, β-bisabolol, and citronellyl tiglate exhibited significant positive correlations with *TPS*2 (IMPMBI2G00000002285, IMPMBI2G00000034968), *TPS*1 (IMPMBI2G00000034867), and IMPMBI2G00000034962. These findings reflect the synergistic regulatory effects of precursor synthesis and downstream catalytic genes on terpene production.

In summary, the differences in terpenoid metabolism among the 3 *MF* varieties are primarily driven by transcriptional-level regulation. The terpenoid biosynthesis pathway and secondary metabolite biosynthesis pathways serve as the core of metabolic specialization across different varieties. The coordinated changes between DEGs in these pathways and key terpenoid metabolites like linalool and (+)-camphor reflect the germplasm-specific regulatory mechanisms underlying the synthesis and accumulation of medicinal active compounds in the 3 *MF* varieties.

### 3.5 Profiles of key candidate genes and metabolites associated with terpenoid metabolisms in 3 *MF* varieties

**Figure 5:**
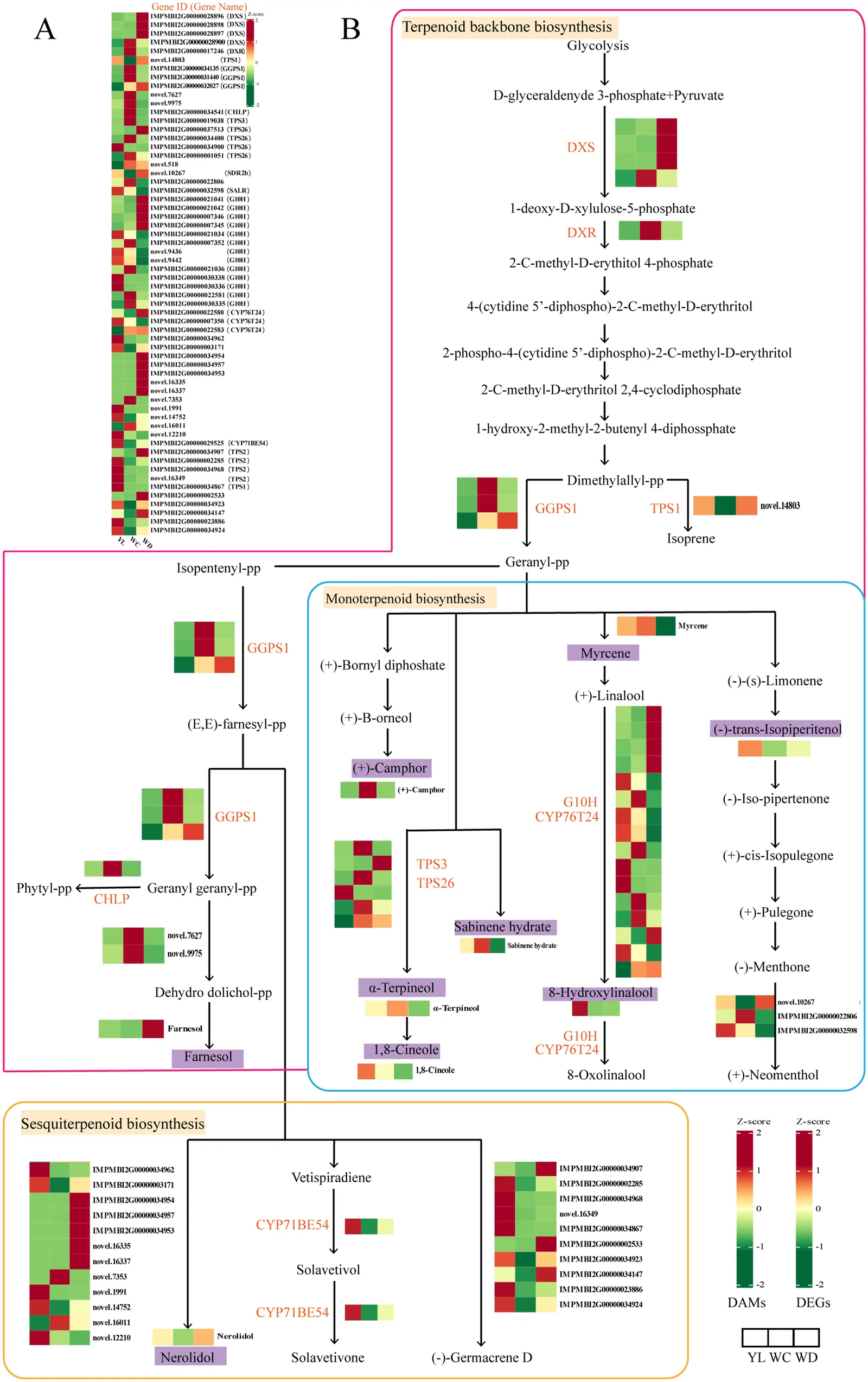
Expression patterns of genes related to the terpenoid biosynthesis pathway in 3 *MF* varieties. **(A)** Relative expression changes in key genes in 3 *MF* varieties. **(B)** Terpenoid biosynthetic pathway.

To better understand the relationship between metabolites and genes in terpenoid accumulation among the 3 *MF* varieties, KEGG pathway enrichment analysis was performed on DAMs and DEGs. A detailed comprehensive analysis was conducted on the genes and metabolites related to the terpenoid biosynthesis pathway. A total of 61 DEGs were co-enriched in the KEGG pathway diagram (**Figure 5A**; **Supplementary Table 6**), including 26 involved in monoterpenoid biosynthesis, 12 in terpenoid backbone biosynthesis, and 23 in sesquiterpenoid biosynthesis. Nine terpenoid differential metabolites were enriched, including (+)-camphor, sabinene hydrate, myrcene, farnesol, 8-hydroxylinalool, (-)-trans-isopiperitenol, α-terpineol, 1,8-Cineole and nerolidol. The integrated pathway results (**Figure 5B**) indicated that several differentially expressed genes exhibited trends similar to those of downstream metabolites. The differential metabolites (+)-camphor, sabinene hydrate, α-terpineol, and myrcene showed significantly higher levels in WC compared to the other two varieties, while the expression patterns of the differential genes *DXR* (IMPMBI2G00000017246), *GGPS*1 (IMPMBI2G00000034135, IMPMBI2G00000031440), *TPS*3 (IMPMBI2G00000019038), and *TPS*26 (IMPMBI2G00000034400) were generally consistent with these changes. In contrast, 1,8-Cineole and 8-hydroxylinalool were more abundant in YL than in the other two varieties, and the regulatory trend of the differential gene *TPS*26 (IMPMBI2G00000034900) was basically consistent with that of 1,8-Cineole, while the regulatory trend of *TPS*26 (IMPMBI2G00000001051) was negatively correlated with 1,8-Cineole. The regulation trends of *G10H* (IMPMBI2G00000030336, IMPMBI2G00000030338) are essentially consistent with 8-Hydroxylinalool, while the regulation trends of *G10H* (IMPMBI2G00000030335) and *CYP76T24* (IMPMBI2G00000022583) are negatively correlated with 8-Hydroxylinalool. The content of Nerolidol in WD is higher than that in the other two varieties, and the regulatory trends of IMPMBI2G00000034953, IMPMBI2G00000034954, IMPMBI2G00000034957, novel.16335, and novel.16337 are generally consistent with it, while the regulatory trend of novel.7353 is negatively correlated with Nerolidol. However, no significant correlation was observed between the expression trends of other DEGs and their corresponding downstream DAMs. This phenomenon is closely related to the fact that terpenoid biosynthesis and expression processes in *MF* involve multi-gene regulation, forming an intricate regulatory network through interactions among multiple substances and genes. Consequently, the relationship does not simply manifest as a one-to-one correspondence between metabolites and genes.

Transcriptomic datasets of *MF* were screened via BLAST homology searches performed in BioEdit. Thresholds of *E*-value < 1e−5 and sequence identity ≥ 50% were applied to filter candidate DEGs, including *TPS*3, *TPS*26 (IMPMBI2G00000037513, IMPMBI2G00000034900, IMPMBI2G00000034400, IMPMBI2G00000001051), IMPMBI2G00000034957 and IMPMBI2G00000034954. Furthermore, TBtools and NCBI ORF finder were used to predict open reading frames. Based on the protein sequences of the identified Nerolidol synthase (Pertea et al., 2015) (GenBank: AQA26342.1) and (-)-α-terpineol synthase (GenBank: AAS79352.1) (Diane et al., 2004) as search models, homology alignment of target sequences was performed using BioEdit software. The results (**Supplementary Figure 3**) showed that *TPS*26 (IMPMBI2G00000037513, IMPMBI2G00000034900, IMPMBI2G00000001051), IMPMBI2G00000034957, and IMPMBI2G00000034954 all contain ‘NSE/DTE’ and ‘DDXXD’ conserved motifs, possess typical terpene synthase catalytic domains, and are speculated to have terpenoid synthesis activity. The conserved motif ‘R(R)X_8_W’ is responsible for the cyclization of monoterpenoids (Williams et al., 1998), but IMPMBI2G00000034957 and IMPMBI2G00000034954 lack this structure, suggesting that their catalytic products are acyclic products, such as geraniol, nerolidol.

The amino acid sequences of the screened candidate DEGs, along with those of known monoterpene synthases and sesquiterpene synthases, were used to construct a phylogenetic tree (**Supplementary Figure 4**) using MEGA 6.0 software with the neighbor-joining (NJ) method and a bootstrap value of 1000. Plant terpene synthases are categorized into seven subfamilies (*TPS*-a to *TPS*-h) according to their protein sequences and functional characteristics. As shown in the figure, the putative artemisinin-like terpene synthase candidate gene *TPS*26 (IMPMBI2G00000037513, IMPMBI2G00000034900, IMPMBI2G00000034400, IMPMBI2G00000001051) belongs to the *TPS*-b clade, which is dominated by cyclic monoterpene synthases. Meanwhile, *SBS* (GenBank: AAC26017) (Mitchell et al., 1998), *LaBPPS* (UniProtKB: C0KWV3.1) (Naoko et al., 2010), AvBPPS (UniProtKB: W6HUT3.1) (Yuechong et al., 2014), *LdBPPS* (GenBank: ATY48638.1) (Matthew et al., 2017), and others also clustered within this branch, suggesting that these candidate genes can cyclize the substrate GPP to generate the target compound monoterpene. IMPMBI2G00000034957 and IMPMBI2G00000034954 are closely related to *CpTPS*4 (GenBank: KAJ8628089.1) (Onkar et al., 2022) and *Lis-LINS* (GenBank: KAH6764609.1) (Yujun et al., 2021), both of which lacked the N-terminal ‘R(R)X_8_W’ motif, a typical feature of the *TPS*-g subfamily. These findings suggest that these genes may play important roles in the accumulation of terpenoids across the 3 *MF* varieties.

### 3.6 RT-qPCR analysis

**Figure 6:**
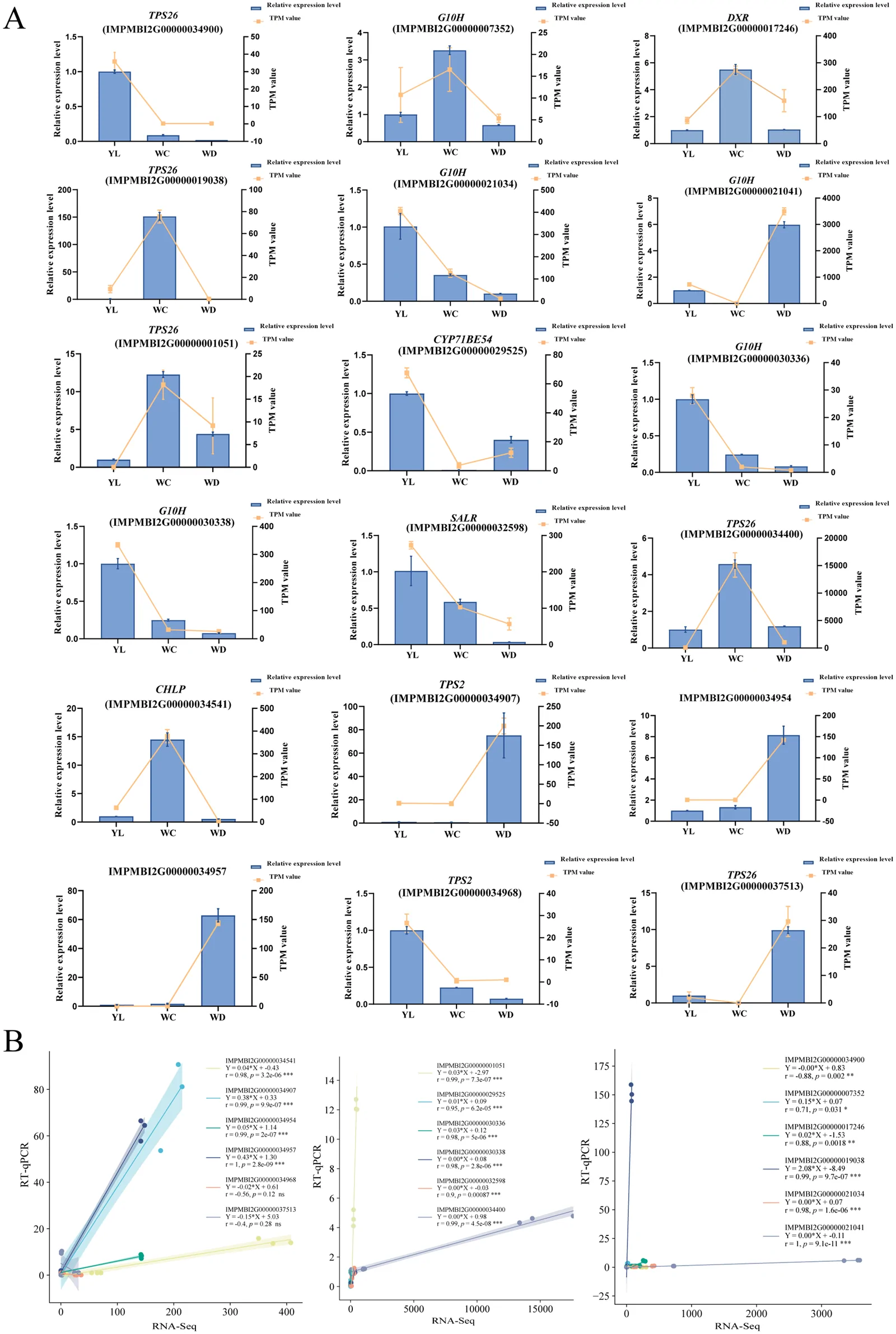
RT-qPCR validation of key biosynthetic genes. The histograms show the RT-qPCR while line chart show TPM values of RNA-seq data. Each sample was subjected to three biological replicates. The error bars represent the standard errors. **(A)** Validation of the expression of gene related to terpenoids. **(B)** The correlation between gene expression levels from RT-qPCR results analysis and RNA-Seq data was assessed using Pearson’s correlation coefficient (*r*) and the sig­nificance was evaluated using a *t*-test.

To validate transcriptomic expression profiles, 18 terpenoid biosynthetic structural genes were subjected to RT-qPCR quantification. Several candidates were prioritized from multi-omics correlation analysis for their tight associations with differential terpenoids. The remaining genes were randomly sampled from terpenoid-related DEGs covering the full biosynthetic cascade to comprehensively verify RNA-seq reliability. The results (**Figure 6A**) showed that *SALR* (IMPMBI2G00000032598) and *G10H* (IMPMBI2G00000021034, IMPMBI2G00000030336, IMPMBI2G00000030338) and IMPMBI2G00000034968 exhibited higher expression levels in YL, followed by WC, with lower expression in WD. *G10H* (IMPMBI2G00000007352, IMPMBI2G00000022581), *CHLP* (IMPMBI2G00000034541), *TPS3* (IMPMBI2G00000019038), *TPS26* (IMPMBI2G00000034400) and *DXR* (IMPMBI2G00000001051) showed the highest expression levels in WC. *TPS26* (IMPMBI2G00000037513), *TPS2* (IMPMBI2G00000034907), *G10H* (IMPMBI2G00000021041), IMPMBI2G00000034957 and IMPMBI2G00000034954 exhibited the highest expression levels in WD, with lower expression in YL and WC. *CYP71BE54* (IMPMBI2G00000029525) and IMPMBI2G00000034900 showed the highest expression levels in YL, followed by WD, and lower expression in WC. The expression patterns of all genes quantified by RT-qPCR in the 3 *MF* varieties were consistent with the sequencing results (**Figure 6B**). These results further supported the potential roles of these genes in the synthesis and regulation of terpenoid metabolites in *MF*.

### 3.7 Analysis of transcription factors related to terpenoid biosynthesis in *MF*

In plants, transcription factors (TFs) regulate the expression of specific genes or a set of genes by promoting or inhibiting the interaction between the RNA polymerase complex and promoters. To explore the transcriptional regulation of terpenoid biosynthesis in *MF*, transcriptome sequences were annotated using the Plant Transcription Factor Database (PlantTFDB). A total of 213 unigenes were annotated as TFs, which were classified into 52 TF families. The top ten transcription factor families included *MYB*, *AP2/ERF-ERF*, *bHLH*, *NAC*, *FAR*1, *WRKY*, *GARP-G2-like*, *MYB-related, C2H2*, and *LOB*, accounting for 12.21%, 11.74%, 7.98%, 7.51%, 5.63%, 5.63%, 4.22%, 4.22%, 3.76%, and 3.29% respectively (**Supplementary Table 7**). Further enrichment analysis of the unigenes from these 52 TF families indicated that only IMPMBI2G00000033291 from the *MYB*-related family was implicated in the regulation of the terpenoid biosynthesis pathway in *MF*. This assignment was based on integrated functional annotation: DIAMOND alignment against KEGG and GO databases identified this unigene as a transcription factor involved in terpenoid biosynthesis (GO:0006721), and its *MYB*-related identity was independently confirmed by iTAK software. The regulation of terpenoid synthesis by TFs may occur through indirect pathways, such as modulating upstream signaling molecules or facilitating synergistic interactions, rather than through direct binding to the promoters of enzyme genes. Consequently, such TFs may not be included in the list of DEGs and may require a regulatory network analysis for their identification. Furthermore, since *MF* is a non-model plant, the PlnTFDB and PlantTFDB may not provide comprehensive annotations for its specific TFs. It is hypothesized that the TFs that actually regulate terpenoid synthesis in *MF* may be species-specific or belong to novel families that have yet to be documented.

## 4 Discussion

As an important traditional Chinese medicinal herb, *MF* relies on its volatile oil components as the core material basis underlying its pharmacological activities, with terpenoids being regarded as key bioactive constituents that have garnered extensive research interest in recent years. However, the biosynthetic pathways and regulatory mechanisms of terpenoids in *MF* remain unexplored. In this study, we employed metabolomic analysis to identify differences in terpenoid compounds among the three *MF* varieties and integrated transcriptomic data to elucidate the expression patterns of terpenoid biosynthesis genes. These findings provided new insights into the metabolism and variety breeding of *MF*, while also offering molecular evidence to support its pharmacological potential.

### 4.1 Differences in terpenoid metabolites of *MF*

Terpenoids, the primary class of compounds found in the volatile oils of *MF*, are recognized as significant contributors to its characteristic aroma and medicinal properties. The content and composition of terpenoid compounds often vary among different plant varieties, leading to differences in pharmacological efficacy. This study utilized metabolomics analysis to clarify the differential metabolic characteristics of three varieties of *MF*. From the distribution of differential metabolites, the WD vs. WC group exhibited the highest number of differential metabolites, while the YL vs. WD group had the least, reflecting the variety specificity of terpenoid metabolism in *MF*. Among the 55 significantly different terpenoids screened, compounds such as linalool, (+)-camphor, citronellol, and citral were found to be highly accumulated in WC, while α-elemol, β-cubene, carveol, and myroxide were predominantly accumulated in YL. Most terpenoids in WD showed relatively low accumulation levels. These results indicate significant differences in the accumulation of terpenoid metabolites among different *MF* varieties, contributing to their unique aromatic characteristics and pharmacological effects. Based on the analysis of the medicinal potential of terpenoids in *MF*, it is speculated that WC and YL may be superior medicinal varieties, providing a basis for the breeding of high-quality magnolia varieties and the study of germplasm resources.

### 4.2 Analysis of the transcriptional regulatory network in terpenoid biosynthesis of *MF*

Currently, the biosynthetic pathways of terpenoid compounds have been largely elucidated, and the cloning, expression and regulation of relevant enzymes involved in this process, as well as their roles in cellular metabolism, have become research hotspots. However, the molecular genetic studies of different varieties of *MF* remain incomplete, which limits the exploration of enzyme genes related to terpenoid synthesis in *MF*. *DXS* is the first key enzyme in the MEP biosynthetic pathway (Tian et al., 2021). Previous studies have shown that the expression level of *DXS* directly affects the accumulation of secondary metabolites in plants (Li et al., 2021a). For instance, in Rose-scented *geranium*, the synthesis of monoterpenes is positively correlated with the *DXS* gene (Jadaun et al., 2016). *DXR* participates in the second step of the MEP pathway and is a rate-limiting enzyme, making it an effective target for regulation (Singh et al., 2007). *GGPS*, a core structural enzyme of terpenoid skeleton formation, catalyzes *IPP* condensation to produce *GPP, GGPP* and *FPP* precursors. This gene has been identified in various plants such as *tree peony* (Li et al., 2021b), *Cryptomeria fortune* (Zhang et al., 2022), and *Rehmannia glutinosa* (Chen et al., 2021), and is associated with the biosynthesis of plant aroma, monoterpenes, sesquiterpenes, and triterpenes. In this study, correlation analysis revealed that monoterpenoid compounds such as linalool and (+)-camphor are significantly positively correlated with genes *DXS*, *DXR*, *TPS*26, and *GGPS1*, indicating that these key enzyme genes play an important role in the upstream synthesis stage of terpenoids and have a significant impact on the synthesis of *MF* terpenoids.

This study integrates the transcriptional regulatory network of terpenoid synthesis, revealing that the downstream metabolite α-terpineol shows a consistent expression trend for *TPS*3 (IMPMBI2G00000019038) and *TPS*26 (IMPMBI2G00000034400) across three different varieties of *MF*, with significantly higher levels in WC compared to the other two varieties. The compound 8-hydroxylinalool and *G10H* (IMPMBI2G00000030336, IMPMBI2G00000030338) exhibit higher concentrations in YL than in the other two varieties, whereas 8-hydroxylinalool and *G10H* (IMPMBI2G00000030335), *CYP76T*24 (IMPMBI2G00000022583) were negatively correlated. In the sesquiterpene synthesis pathway, the downstream metabolite nerolidol, along with IMPMBI2G00000034953, IMPMBI2G00000034954, IMPMBI2G00000034957, novel.16335, and novel.16337, demonstrates higher levels in WD compared to the other two varieties; however, the expression trends of nerolidol and novel.7353 show a negative correlation.

This study conducted homology alignment based on the identified protein sequences of *NES* and (-)-α-terpineol synthase, revealing that *TPS*26 (IMPMBI2G00000037513, IMPMBI2G00000034900, IMPMBI2G00000001051), IMPMBI2G00000034957, and IMPMBI2G00000034954 possess typical terpene synthase catalytic domains. They are speculated to have terpenoid synthesis activity, and further functional verification is required.

Phylogenetic analysis (**Supplementary Figure 4**) classified the candidate *TPS* genes into distinct subfamilies correlating with chemotypic divergence. *TPS*26 members clustered within the *TPS*-b clade (cyclic monoterpene synthases) and were predominantly expressed in WC, coinciding with the highest α-terpineol accumulation. Conversely, IMPMBI2G00000034957/34954 lacked the RRX_8_W motif, grouped with *TPS*-g members, and peaked in WD alongside nerolidol. While YL showed moderate *TPS*26, it exhibited pronounced *G10*H upregulation and the highest 8-hydroxylinalool, indicating that monoterpene oxidation dominates the YL chemotype. The retention of paralogous *TPS* copies with subfamily-level partitioning is consistent with neofunctionalization following gene duplication (Feng et al., 2011). Notably, such *TPS* subfamily partitioning linked to varietal terpenoid divergence has not been previously documented in *Magnoliaceae*; our findings thus establish the first evidence linking *TPS* paralog diversification to the “medicinal material basis” divergence among traditional *MF* cultivars.

However, there is no significant correlation between the changes in other DEGs and their downstream differential metabolites in this study. This phenomenon is relatively common in the research of secondary metabolite regulation in medicinal plants and can be analyzed from multiple mechanistic perspectives. Firstly, the biosynthesis of terpenoids is a complex process involving multiple steps and the coordinated participation of various enzymes (Niu et al., 2015). In addition to the core synthetic enzyme genes, it also involves the co-regulation of metabolite transport protein genes, modification enzyme genes, and degradation-related genes. This synergistic action of a multi-gene network breaks the one-to-one correspondence between single genes and metabolites. Furthermore, the transcriptional regulatory network appears sparse and indirect. Although a *MYB*-related TF (IMPMBI2G00000033291) was identified via functional annotation and iTAK prediction, the limited sample size precluded robust co-expression analysis. The absence of widespread TF-biosynthetic gene correlations suggests that terpenoid regulation in *MF* follows a non-linear model, likely involving upstream signaling modulation or combinatorial interactions rather than direct promoter binding.

Secondly, the existence of post-transcriptional regulatory mechanisms may lead to inconsistencies between gene expression levels and final enzyme activity. For example, miRNA-mediated gene silencing, protein phosphorylation modifications, can cause differences at the mRNA level to not directly reflect on metabolite accumulation (Anurag et al., 2023). Furthermore, the inter-organelle transport and intercellular distribution processes of terpenoid metabolites are regulated by transporter genes, whose expression patterns are independent of synthase genes, also resulting in insignificant correlations between genes and metabolites. At the same time, the genetic background differences among different varieties of *MF* may lead to specific differentiation of the regulatory network. For instance, interspecific differences in the binding efficiency of transcription factors to target genes may result in varying regulatory effects of the same synthetic enzyme gene on metabolites across different varieties. This is consistent with the variety-specific correlation patterns observed in the expression of phenolic synthesis genes and the accumulation of metabolites among different varieties of *Perilla frutescens* (Hong et al., 2025).

### 4.3 Limitations and future perspectives

This study integrated transcriptomic and GC-MS data with six valid biological replicates per cultivar, providing robust statistical support for screening DEGs and differential terpenoid metabolites. Nevertheless, several notable limitations exist in the present work.

First, all regulatory deductions are exclusively based on correlation analysis between gene expression levels and terpenoid accumulation. Strong covariation only reflects synchronized transcriptional and metabolic shifts across varieties, and such correlations cannot be taken as definitive causal regulatory evidence without additional functional experiments. Second, only RT-qPCR was applied to verify transcriptional expression patterns; we have not performed *in vitro* enzyme activity assays or plant transgenic functional tests to directly confirm the catalytic capacity of candidate *TPS* proteins. For subsequent research, *in vitro* enzyme activity detection and transient plant transformation assays will be carried out to characterize the catalytic functions of core *TPS* genes, establishing direct causal evidence connecting gene expression with terpenoid biosynthesis. Large-scale field germplasm screening can also be conducted to translate the identified metabolic markers into practical *MF* quality evaluation and breeding applications.

## 5 Conclusion

This study systematically revealed the terpenoid metabolic differences and molecular regulatory mechanisms among three varieties of *MF* through integrated metabolomics and transcriptomics analysis. Metabolomic screened 55 significantly differential terpenoids, among which 15 core compounds could act as characteristic metabolic markers for discriminating different *MF* varieties. Transcriptional network analysis of terpenoid biosynthesis indicated that *TPS*3 (IMPMBI2G00000019038), *TPS*26 (IMPMBI2G00000034400), and *G10H* (IMPMBI2G00000030336, IMPMBI2G00000030338) represented key candidate genes responsible for monoterpene biosynthesis in *MF*. Furthermore, several genes, including IMPMBI2G00000034953, IMPMBI2G00000034954, IMPMBI2G00000034957, novel.16335, and novel.16337, were predicted as core candidates participating in *MF* sesquiterpene biosynthesis. These genes were closely correlated with metabolite accumulation patterns, suggesting their essential contributions to varietal terpenoid divergence. The expression patterns of these candidate genes displayed strong correlations with the accumulation profiles of differential terpenoids across varieties, which reflects coordinated transcriptional-metabolite variation rather than definite causal regulatory effects. Homology alignment of protein sequences demonstrated that *TPS*26 (IMPMBI2G00000037513, IMPMBI2G00000034900, IMPMBI2G00000001051), IMPMBI2G00000034957 and IMPMBI2G00000034954 harbored canonical terpene synthase catalytic domains and presumed terpenoid biosynthetic activity, which requires further functional verification. Additionally, RT-qPCR validation confirmed the expression trends of several key candidate genes, thereby enhancing the reliability of the transcriptomic data. In summary, this research elucidated the molecular mechanisms underlying terpenoid metabolism in *MF*, offering a theoretical foundation for selective breeding and improving medicinal quality through metabolic engineering.

## Data Availability

The raw sequence data reported in this paper have been deposited in the Genome Sequence Archive (Genomics, Proteomics & Bioinformatics 2025) in National Genomics Data Center (Nucleic Acids Res 2025), China National Center for Bioinformation/Beijing Institute of Genomics, Chinese Academy of Sciences (GSA: CRA037470) that are publicly accessible at https://ngdc.cncb.ac.cn/gsa.
