# Supplementary material for: Integrative analyses of metabolome and transcriptome reveal differences in terpenoids and identify the genes involved in the synthesis of terpenoids in Magnoliae Flos from different varieties
Source: Front Plant Sci. 2026 Aug 26;17:1894544. doi: 10.3389/fpls.2026.1894544 (PMC13553886; doi:10.3389/fpls.2026.1894544)
Supplement: Supplementary file 1 [file DataSheet1.docx]

## 1.1 Supplementary Figures

Figure. S1.WC vs YL vs WD OPLS-DA_permutation. WC: *Magnolia biondii* Pamp.), YL: *Magnolia denudata* Desr.), WD: *Magnolia sprengeri* Pamp. The names and abbreviations corresponding to Figure.S2-S4 are the same as above.

Figure.S2.WC vs YL vs WD OPLS-DA_scorePlot.

Figure.S3. Alignment of the amino acid sequences of key candidate genes. The protein sequences were aligned using BioEdit. The conserved RRX_8_W, DDXXD, and NSE/DTE domains are underlined.

Figure.S4. Phylogenetic analysis of key candidate genes. The tree was constructed using the neighbor joining method in MEGA 6.

Figure.S5 Total ion current chromatogram (TIC) of mixed quality control (QC) samples. The x-axis represents the retention time (Rt) of metabolite detection, and the y-axis represents the ion current intensity of ion detection (with intensity unit in cps, count per second).

**Supplementary Table titles:**

Supplementary Table S1.Sample information of *Magnoliae Flos*.

Supplementary Table S2. Primers for RT-qPCR.

Supplementary Table S3. K-means clustering of terpenoid metabolitesxlsx.

Supplementary Table S4. 55 DAMs in 3 *MF* varities.

Supplementary Table S5. Summary of reads quality after sample Illumina sequencing.

Supplementary Table S6. Related differential metabolites and genes involved in terpenoids biosynthetic pathways, along with their expression levels

Supplementary Table S7.Transcription factors related to terpenoid biosynthesis in *MF*.

Supplementary Table S8. All sample data of *MF*.
